# Barriers inhibiting effective detection and management of postpartum hemorrhage during facility-based births in Madagascar: findings from a qualitative study using a behavioral science lens

**DOI:** 10.1186/s12884-021-03801-w

**Published:** 2021-04-22

**Authors:** Sara V. Flanagan, Tina Razafinamanana, Charlotte Warren, Jana Smith

**Affiliations:** 1 ideas42, 80 Broad St Fl 30, New York, NY, 10004 USA; 2 Population Council, 4301 Connecticut Avenue NW, Washington, DC, 20008 USA

**Keywords:** Maternal mortality, Postpartum hemorrhage, Healthcare provider, Madagascar, Facility-based birth, Behavioral barriers

## Abstract

**Background:**

Postpartum hemorrhage (PPH) is the leading cause of maternal mortality in low-income countries, and is the most common direct cause of maternal deaths in Madagascar. Studies in Madagascar and other low-income countries observe low provider adherence to recommended practices for PPH prevention and treatment. Our study addresses gaps in the literature by applying a behavioral science lens to identify barriers inhibiting facility-based providers’ consistent following of PPH best practices in Madagascar.

**Methods:**

In June 2019, we undertook a cross-sectional qualitative research study in peri-urban and rural areas of the Vatovavy-Fitovinany region of Madagascar. We conducted 47 in-depth interviews in 19 facilities and five communities, with facility-based healthcare providers, postpartum women, medical supervisors, community health volunteers, and traditional birth attendants, and conducted thematic analysis of the transcripts.

**Results:**

We identified seven key behavioral insights representing a range of factors that may contribute to delays in appropriate PPH management in these settings. Findings suggest providers’ perceived low risk of PPH may influence their compliance with best practices, subconsciously or explicitly, and lead them to undervalue the importance of PPH prevention and monitoring measures. Providers lack clear feedback on specific components of their performance, which ultimately inhibits continuous improvement of compliance with best practices. Providers demonstrate great resourcefulness while operating in a challenging context with limited equipment, supplies, and support; however, overcoming these challenges remains their foremost concern. This response to chronic scarcity is cognitively taxing and may ultimately affect clinical decision-making.

**Conclusions:**

Our study reveals how perception of low risk of PPH, limited feedback on compliance with best practices and consequences of current practices, and a context of scarcity may negatively affect provider decision-making and clinical practices. Behaviorally informed interventions, designed for specific contexts that care providers operate in, can help improve quality of care and health outcomes for women in labor and childbirth.

**Supplementary Information:**

The online version contains supplementary material available at 10.1186/s12884-021-03801-w.

## Background

Madagascar’s total fertility and maternal mortality rates have remained high, with an average of 5.0 children per woman [1] and 353 deaths per 100,000 live births [2], respectively. Madagascar’s population, estimated at 27 million in 2019 [3], is largely rural, and although 82% of pregnant women receive at least one antenatal care (ANC) contact, only 44% of births are attended by a skilled health care provider, with 38% of deliveries in a health facility [4]. While the majority of births currently take place outside of facilities, improving rates of institutional delivery [4] suggests that facility-based providers will play an increasingly important role in reducing maternal mortality from postpartum hemorrhage (PPH).

PPH is the leading cause of maternal mortality in low-income countries [5]. A national assessment of 303 health facilities in Madagascar found that direct causes contributed to 84% of maternal deaths reviewed, with PPH the most common direct cause, followed by pre-eclampsia/eclampsia, obstructed labor, and infection [6]. Active management of the third stage of labor (AMTSL), of which administration of a uterotonic immediately after birth is a central component, remains the primary strategy for PPH prevention recommended by the World Health Organization (WHO) [7]. Oxytocin is recommended as the first uterotonic of choice and plays a central role in the treatment of PPH.

Although AMTSL for every birth is national policy in Madagascar [8], steps for correctly performing it may not be followed consistently during deliveries. Studies of provider AMTSL guideline implementation in low-income countries often find high adherence to individual components of AMTSL, but less for all components [9–12]. A 2011 survey of 36 health facilities in Madagascar by the Maternal and Child Health Integrated Program (MCHIP) found that oxytocin was given in 85% of deliveries observed, but it was only given within 1 min of birth in the correct dose and route in 21% of cases. In the same study, compliance with all elements of AMTSL occurred in only 13% of observed deliveries [13], and in the few instances of PPH complications observed during delivery, uterotonics were only administered in half of those observed cases, despite uterotonic availability in the facilities [13].

Identified barriers to adequate implementation of evidence-based interventions recommended by WHO guidelines such as AMTSL range from healthcare system and supply issues, to insufficient knowledge due to lack of awareness or access to guidelines, in addition to numerous contextual and interpersonal influencing factors [9, 12, 14]. However, these assessments rarely focus on the psychological drivers of health care provider behavior and how their environments, including structural constraints, physical context, and social influence, may shape how they provide care and their adherence to known clinical protocols.

Our study addresses this discrepancy in the literature by employing a behavioral science lens to identify barriers that inhibit facility-based providers from following best practices for PPH prevention and management in Madagascar. Insights from behavioral science can help develop a more nuanced understanding of provider decision-making and behavior related to obstetric complications and identify opportunities for innovative solutions to address barriers faced in providing quality care, specific to the challenging circumstances in which providers work.

## Methods

### Study aim and design

The aim of this study was to identify behavioral barriers that may inhibit facility-based providers in Madagascar from consistently following best practices for PPH prevention and management during childbirth. A second aim was to identify features of their context shaping their behavior that can be addressed to develop solutions to this problem. To understand the barriers providers face, we conducted a cross-sectional qualitative research study with in-depth interviews with providers as well as key stakeholders who may influence, or have a perspective on, providers’ behavior. This formative research served as the diagnosis stage of our behavioral design process [15] for program development.

### Study setting

Study sites included 2 CHRDs, 17 CSBs, and five communities in peri-urban and rural areas of Vohipeno and Manakara districts in the Vatovavy-Fitovinany region of Madagascar. Sites were selected at the recommendation of our local partner and based on high rates of PPH in this region [16]. In Madagascar, Basic Health Centers (CSBs) are equipped to attend women in labor and childbirth while District Hospitals (CHRDs) provide the next level of care. Remote CSBs are often run by a nurse or midwife rather than a doctor, in practice if not officially, often without electricity or running water, and primarily deliver outpatient care. Fieldwork was conducted during 2 weeks in June 2019.

### Study participants

Study participants included: facility-based healthcare providers (*n* = 26) who attend deliveries, including doctors, midwives, nurses, trainees, and medical supervisors; postpartum women (*n* = 11) that had delivered within the previous 6 months, who, in some cases, had experienced bleeding complications; community health volunteers (CHVs) (*n* = 7) who liaise between health facilities and their communities; as well as traditional birth attendants (*n* = 3), referred to locally as matrons.

### Processes

A convenience sample of participant groups represented a range of perspectives on childbirth complications. To develop interview guides, we first drew up a detailed process map of clinical steps generally considered as best practice for prevention and detection of complications in routine deliveries, as well as specific best practices for PPH management, and validated the list of behaviors with partners and a regional clinician. After finalizing the process map, we then systematically generated hypotheses about behavioral barriers that facility-based providers may face in attempting to follow best practices for PPH detection and management during childbirth, and the factors in the environment that might contribute to these barriers. Hypotheses arose out of a series of organizationally-developed question prompts, which cover different psychological principles and insights from behavioral sciences, and reviews of other studies on related provider behaviors in resource-limited settings [15, 17]. Hypotheses sought to explain why providers who have been trained on best practices for PPH prevention and management might fail to follow them consistently. In-depth interviews (IDIs) with providers and supervisors covered their backgrounds, professional responsibilities, dynamics within their facilities, typical practice during labor and delivery, experiences with and responses to complications, and interactions with patients and families. The final research tools (see Supplementary) were tested with healthcare providers in Antananarivo.

Although the focus of this study is on facility-based providers, IDI guides were developed for other stakeholder groups to explicitly solicit different perspectives on their behavior related to PPH complications. These varied perspectives helped to generate a greater understanding of how different contextual figures and features may ultimately influence provider behavior. District health officials, facility-based providers, and local community mobilizers helped recruit postpartum women, CHVs, and matrons within communities for interviews. Several postpartum women interviewed were attending the health facility for immunizations and gave consent for an interview that same day. Recruitment continued until investigators felt they had achieved saturation of information and perspective. Participants received 5000 ariary (approximately $2.50) as compensation for their time. Interviews with postpartum women included questions on their background, birthing experience, provider interactions, and both the positive and negative elements of their experience. Interviews with CHVs covered their backgrounds, volunteer responsibilities, perceptions of both providers and patients, and perspectives on local women’s experience with delivery and complications. Interviews with matrons covered their background, professional responsibilities, typical practices during labor and delivery, experience with and responses to complications, interactions with women and their families, and interactions with health facilities and facility-based providers.

Interviews were conducted by two female non-clinical public health and social science researchers, one local Malagasy and one foreign non-Malagasy, with simultaneous interpretation for the non-Malagasy researcher. Participants were interviewed only once; the investigators and interpreter had no relationship with participants prior to conducting the interviews. We administered a voluntary, informed consent process with each participant, with written consent obtained before beginning any interviews, which were conducted in private and audio recorded. We obtained permission from facility supervisors to speak with their staff. Investigators took written notes of their own personal reactions during the recorded interviews and debriefed on a daily basis during data collection. Our research protocol was approved by Population Council’s Institutional Review Board (as Protocol #897) and the Madagascar Ministry of Public Health Committee on Research Ethics.

### Analysis

English interpretation of IDI recordings were transcribed verbatim. Interviews fully conducted in Malagasy were audio-recorded and then simultaneously translated during transcription so that live features of the recording, such as intonation and pauses, were considered when translating into English. Interview interpretation and translation services were provided by a single certified translator, native to the study region, who is familiar with local dialects, accents, and culture. We then conducted thematic analysis of interview transcripts [18]. Using the hypothesized barriers and contextual features, and amending and adding themes as they emerged, we developed a coding framework through which members of the research team individually coded each transcript. Several transcripts were also double-coded to ensure sufficient agreement and consistency among researchers. Once all interview transcripts were coded, data relevant to each hypothesis was excerpted and charted based on the final thematic framework. The research team then individually assessed whether the preponderance of evidence across participant groups supported or contradicted each hypothesis, resolving any discrepancies through discussion until consensus. This process elevated the most relevant behavioral barriers and contextual features based on the strength of available evidence, with consideration for support in other behavioral literature, to generate the key insights into provider behavior reported here. Each of these insights suggests a mechanism linking an identified barrier and its relevant contextual features to poor provider adherence to PPH best practices. Reflexivity was maintained by the research team at all stages of analysis through continuous dialogue to challenge assumptions and allow for new insights to emerge. Summary findings and interpretations were validated with local partners and informally checked with providers during subsequent solution co-creation activities. This manuscript follows O’Brien et al. Standards for Reporting Qualitative Research [19].

## Results

Maternal health-care providers interviewed ranged from nurse and midwife trainees only recently employed, to doctors and midwives with more than a decade of experience. Interviews with providers and other stakeholders familiar with their working context revealed seven specific insights about behavioral barriers inhibiting providers’ consistent adherence to best practices for PPH, in addition to important contributing features from their environment. Although not all of these insights apply to all providers, they represent a range of factors contributing to delays in PPH detection or ineffective management in the study settings.

1. Due to perceived low prevalence of PPH, providers focus on risks of other delivery complications, which leads to their undervaluing of the importance of strict compliance with preventive measures for PPH.

Providers most commonly mentioned being on the lookout for signs that they would need to refer due to a complicated or prolonged labor, such as the position or size of the fetus, the mother’s condition – e.g. whether she is “tired” or having convulsions, or difficulty in getting first-time mothers to push “correctly,” a delay which they feel could endanger the baby. Such complications often cannot be managed at CSBs, and providers face significant difficulty in securing patient cooperation for timely referral to higher level facilities, mostly due to financial and transportation challenges. When explicitly asked about PPH, most providers reported minimal or no experience managing women with PPH. Due to the perceived low probability of PPH in comparison to other complications, providers may in turn believe strict adherence to PPH preventive measures, such as timely oxytocin administration during AMTSL (GATPA in French), is of less importance. Findings suggest that the benefits of PPH prevention are undervalued in relation to other provider priorities and demands on their attention. Due to the low perceived risk of PPH, providers who often must charge patients for oxytocin when project-funded supplies of uterotonics are exhausted may decide to circumvent clinical protocols to avoid having to convince families that oxytocin is worth paying for. Two nurses interviewed admitted not using the GATPA process on multiparous women they perceive at less risk; one explained that it is often to save their patients the expense of oxytocin:

*“If I haven’t used oxytocin yet, then I don’t use the GATPA process … In fact, people usually don’t have money, so you do all the possibilities not to make them pay.”* (Nurse)

2) It is difficult to know if one is applying oxytocin within the 1 min window, and the difference between applying within 1 min and soon after is not often apparent; thus providers apply oxytocin after the minute window without realizing it.

While providers often reported use of oxytocin during the GATPA process as routine clinical practice after delivery, none correctly described the precise, recommended timing of its administration, even when probed. This could imply a knowledge gap, or that the importance of timing is of less concern for providers not explicitly thinking about the consequences of delayed administration. Providers’ descriptions of the various tasks related to immediate newborn care happening prior to GATPA suggest that administration of oxytocin typically occurs much later than 1 min after birth, as observed in previous facility assessments [13].

*“We do the clamping, and we cut the umbilical cord about three centimeters. We clean the baby and prepare the clothing. And, after five minutes, we check the mother’s bleeding. And we check the placenta and at the end we use the oxytocin.”* (Midwife)

With little or no experience with PPH cases, providers cannot compare what happens when they apply oxytocin immediately after birth with delayed application. Furthermore, we found that since there is no visible clock or other feedback measure to appreciate the magnitude of delay in administration, providers are not aware that they are not following best practice.

3) Very few women present with PPH; therefore, providers’ default assumption is that there will not be cases of PPH, so they do not systematically estimate blood loss until visible cues tell them otherwise, thus delaying PPH detection.

When asked to describe routine care, few providers reported monitoring blood loss, much less systematic measurement. When asked about PPH detection, providers reported excessive blood on white fabric as the visual cue that PPH is present.

*“Sometimes it bleeds a lot and we have the white fabric that we put there and it fills very quickly and we clean up and use another one. It fills very quickly, then we know that it’s risky.”* (Trainee midwife)

These comments highlight that providers operate on the assumption there will be no PPH and expect clear and obvious signs of bleeding to alert them otherwise.

4) Providers may rely on family members or clients to tell them if there is bleeding rather than monitoring it themselves, especially if they are working alone or the delivery is late at night, which may delay PPH detection.

Our findings suggest that perception of low risk of PPH may contribute to lack of consistent post-partum monitoring that can delay PPH detection.

*“The patient is there [at the CSB] and they [the staff are] going to sleep for some time. They come back, and then they discover [PPH].”* (Supervisor)

When providers are unable to check on clients regularly after delivery, or believe it isn’t necessary, they may rely on patients or their families to tell them if there is bleeding.

*“No he [the doctor] didn’t [ever check bleeding]. Because they were just asking questions on the blood but actually didn’t care about it. I think he could have just checked and do something, but he did not.”* (Postpartum woman)

*“And I told the person who was with her to look after her and call me if she is bleeding, but that person didn’t and when I passed to examine her again, she had a hemorrhage and I had to re-check what happen to her.”* (Midwife)

Despite recognizing the risk of relying on women and their family members to detect and report bleeding, exhausted midwives convinced that the likelihood of complications is low, or if they are operating in a situation of scarcity [20], may narrowly focus on the risks most salient to them – most likely other women currently in labor rather than the woman who has already delivered.

5) Providers have a mental model of hemorrhaging as rapid, extreme blood loss; therefore, providers may discharge patients with slow, continuous blood loss and fail to diagnose them with PPH.

Both the experiences and expectations of bleeding that providers described are consistent with a mental model [21] of PPH as rapid, extreme blood loss. When probed, providers could rarely recall ever having seen or heard of a case of PPH with slow but continuous bleeding. Providers frequently remarked that women and their families are often eager to leave the facility and at times there is pressure to free up limited space and beds, creating added incentives for early discharge.

*“The least I can do is keeping them for three, four hours. When they’re fine, then I would let them go home.”* (Midwife)

This tendency to assume there is no PPH without heavy bleeding may lead to missed cases, along with premature patient discharge, which could result in undocumented deaths in the community.

*“There might be some cases [of PPH in the community]. Because we don’t know.”* (Doctor)

Without clear feedback on cases of PPH occurring in a community, providers are not made aware of the consequences of early patient discharge, preventing reflection that could lead to behavior change.

6) Even if trained in PPH management, the small number of PPH cases that most providers encounter makes it easy to forget what one should do, especially with most facilities lacking easily accessible and understandable visual reminders of clinical procedures, or anyone to ask at the moment of action.

Most providers interviewed had seen few, if any, women with PPH outside of training, and had difficulty describing the steps they would take to manage such a condition. Given the low volume of deliveries in many facilities, limited instances of PPH, and variations in PPH treatment protocol depending on source, it is unlikely that providers will recall what to do in the moment complications arise, despite their training.

While posters with clinical algorithms were posted in some facilities, these materials were not available in more remote CSBs where they might be particularly important because a timely referral during a critical situation would be challenging to realize. Even providers in peri-urban facilities struggled to explain how they would proceed with PPH management using the poster, suggesting the content is not intuitive or clear. Several providers reported that they would refer to books in complicated cases, but in a situation with heavy bleeding when the provider is alone, it is difficult to envision how a provider would easily be able to refer to a book in order to know how to proceed.

Given the lack of clear reminders or accessible reference materials, and with no one to ask for help, most providers follow their intuition as to what they should do.

*“I decided on my own when I was in the countryside … I was the only one at the CSB II, no doctor, no one else but me.”* (Midwife)

One midwife described the only time she had seen PPH, in which she tried putting her hand inside the uterus to stop the bleeding:

*“Because I found that the source of the bleeding was from the interior, so I had to find a technique, that’s how I concluded with my fist like that to stop the bleeding.”* (Midwife)

7) There is a mismatch between the practices providers are trained on and what is feasible in the facilities, requiring providers to adapt best practices according to their own experience or based on what others at a facility tell them should be done.

Most providers receive training on best practices for complications management during their academic preparation but receive limited practical experience applying them until they work in health facilities. Providers shared that when placed in facilities, they were exposed to situations and constraints, such as lack of critical materials, that did not come up during training.

*“Even though we had training practices, it was not enough … Most of the things that we have learnt at school are not useful while we work.”* (Doctor)

Others remarked that under these circumstances, they look to others in their facility to understand how practices are adapted.

Providers also described needing to adapt their practice to the material constraints of rural facilities, remarking that they lacked the necessary equipment and medicines to practice according to their training. Essential commodities and medicines for PPH management are often unavailable, and providers shared they read leaflets of medicines they have on hand to determine potential alternatives. We found evidence that this tendency, at times, leads providers to apply practices outside of protocol. For example, some providers reported using vitamin K1 for PPH management since one of the medical indications mentions blood clotting (albeit for newborns).

*“I just wait for the bleeding to stop, but I give K1 vitamin after delivery, because it helps with the hemorrhage … which I don’t know exactly how as I haven’t had any training.”* (Nurse)

Tranexamic acid, now recommended by WHO for use within 3 h of birth in addition to standard PPH care [22], is not a treatment that providers had heard of.

## Discussion

Our study advances prior research on provider adherence to PPH best practices by exploring dimensions of quality of care through a behavioral lens. Findings support the need to facilitate providers’ optimal clinical decision-making and consistent application of best practices in order to improve outcomes for women experiencing complications. Our research suggests that these gaps may be addressed with thoughtful behavioral solutions that go beyond training and information provision and respond to the challenging conditions in which many facility-based providers in Madagascar work.

Risk perception is a key component of self-protective health behavior models, and has been explored in the global health literature for topics such as pregnancy and HIV risk related contraceptive use [23, 24]. Less explored is risk perception in the realm of provider behavior and quality of care, particularly in low and middle-income countries. Our findings highlight that providers’ perceived low risk of PPH among their patients ultimately influences their adherence to best practices, either subconsciously or explicitly. This aligns with evidence that personal beliefs about PPH risk can lead providers to selectively follow guidelines for prophylactic uterotonic use [25]. While PPH prevalence may be higher in the study districts relative to others in Madagascar, and the rates of PPH in Madagascar may be higher relative to other countries, behavioral science points to the importance of understanding a decision-maker’s true reference point [26] as the lens through which decisions are made. A provider in a rural facility, without intervention, is unlikely to estimate PPH risk by comparing local rates to other settings. Our research suggests that providers’ risk calculation, even if never explicit, instead weighs PPH prevalence relative to other complications personally encountered in the past or relayed by others. These points of reference can subconsciously lead to undervaluation of the importance of PPH prevention and monitoring measures, leading to inconsistent practice and delayed detection.

Another behavioral insight supported by our findings relates to lack of clear feedback on specific components of performance, which ultimately inhibits continuous improvement of compliance with best practices related to PPH, especially the case in the absence of consistent formal supervision. Psychological research has long established the role that feedback on performance and progress towards goals has on behavior [27]; moreover, evidence supports that feedback can improve clinical performance specifically [28, 29]. Our research highlights, for example, barriers to providers’ consistent, timely administration of oxytocin without feedback on discrepancies between their performance and best practice, or without experiencing direct visible consequences due to their delayed application. This is in part due to the small number of avoidable cases, making it difficult to detect a difference between protocol compliance and not. Furthermore, because many providers perform deliveries alone, our findings suggest they can face a trade-off between timely administration of oxytocin and immediate newborn care, requiring providers to prioritize complying with one protocol over another without any formal guidance [30]. Additionally, feedback on patient outcomes in the wider community, particularly missed cases of PPH, which could make the consequences of early discharge more salient, may help to reframe providers’ perceptions of PPH risk as it relates to their post-partum monitoring practices.

While scarcity of time and resources affects a provider’s ability to comply with best practices, behavioral research also shows that a context of scarcity may ultimately affect clinical decision-making [20, 31]. We found that while providers demonstrate great resourcefulness in addressing structural challenges that impede their work, overcoming these challenges on their own is of foremost concern and likely limits their ability to appropriately respond to and manage complex cases of PPH**.** Interviews suggest these challenges range from stock-outs of important commodities and medicines and lack of electricity in facilities, to pressure to charge low-income clients for services and materials or to convince family members to travel long distances at great expense for higher-level care after complications arise. Behavioral science research suggests that navigating a context of scarcity taxes cognitive bandwidth [20], leading individuals to narrow their attention to a limited set of concerns and hindering complex decision-making outside those areas of focus [32]. Insights from this body of research suggest that clinical decision support [33] tailored to this context could facilitate optimal provider behaviors for PPH management, when cognitive bandwidth is taxed and PPH is not top of mind. Given the low case volume of PPH at an average rural facility (which may only see four births per month), real-time decision support may be crucial, as training-acquired knowledge is unlikely to be converted into habit if there are limited opportunities for practice.

To further reduce maternal mortality, significant progress must be made in PPH prevention, timely detection, and appropriate management. Studies in Madagascar and other developing countries have observed limited provider adherence to recommended practices for PPH prevention and treatment during facility births, under a range of working conditions [6, 9–14]. While addressing structural challenges may alleviate common stressors in a provider’s working environment and improve quality of care, it will not eliminate observed discrepancies in provider adherence to best practices. Similarly, interventions that rely on training as a primary means for improving provider adherence to best practices are likely to fall short. Clinical skills training alone will not address underlying beliefs about PPH prevalence, and content conveyed in a singular training is unlikely to translate into the strengthened clinical intuition necessary for optimal performance in challenging contexts and situations.

Several limitations apply to this study. Our interview findings may be subject to bias due to how questions were asked or the identities of the researchers conducting the interviews. To address this, interview guides were vetted for biased or leading questions and interviews with all participant groups were conducted by both a Malagasy and non-Malagasy researcher; we did not find significant differences in responses offered to each interviewer. Another limitation was that project constraints limited time spent in the field and interaction with participants during data collection, which challenged our ability to assess reflexivity. However, we relied on investigator triangulation during data collection and analysis as well as validation with local stakeholders to strengthen the trustworthiness of findings. While the broader concepts behind these behavioral insights may be generalizable to facility-based providers in comparable contexts, the findings presented here are specific to our study population. Early plans to include an ethnographic assessment within delivery wards during the study were canceled because the local IRB did not approve observations of clinical care. Without an opportunity to observe provision of care directly, especially during cases of PPH, we have a more limited understanding of providers’ decision-making context in the moment. To address this limitation, we sought to triangulate descriptions of providers’ clinical decisions and in what context from several different perspectives, including post-partum women, CHVs, matrons, and medical supervisors. Descriptions of providers’ working context presented in this study reflect these multiple perspectives.

## Conclusions

This study’s behavioral science lens identifies barriers inhibiting providers’ consistent best practices for PPH prevention and management in Madagascar that are not represented in the current literature. Our findings illustrate how low risk perception of PPH, limited feedback on compliance with best practices and the consequences of current practices, and a context of scarcity can all negatively affect provider decision-making and clinical practice for PPH prevention and management. Behaviorally informed interventions, designed for the exigencies and specific contexts of care provision, will help improve quality of care and health outcomes for women in labor.

## Supplementary Information

**Additional file 1.** Conversation guides for interviews with providers, supervisors, postpartum women, and community health workers.

## Data Availability

The datasets generated and analyzed during the current study are not publicly available since publicizing them could compromise study participants’ privacy and anonymity, but are available from the corresponding author on reasonable request.

## Abbreviations

AMTSL: Active management of the third stage of labor
ANC: Antenatal care
CHRD: District hospitals (Centres Hospitalier de Référence du District)
CHV: Community health volunteer
CSB: Basic Health Centers (Centres Santé de Bases)
GATPA: Gestion active de la troisième période de l’accouchement
MCHIP: Maternal and Child Health Integrated Program
PPH: Postpartum hemorrhage
WHO: World Health Organization

## References

[1] INSTAT. Madagascar millenium development goals national monitoring survey, 2012-2013, ENSOMD report summary. Antananarivo: INSTAT; 2013. Available at: https://madagascar.unfpa.org/sites/default/files/pub-pdf/OMD_Summary_0.pdf.

[2] WHO (2015). Levels and trends for maternal mortality: 1990 to 2015.

[3] UNDP. World population prospects: the 2019 revision: United Nations Population Division; 2019.

[4] UNICEF (2014). State of the world’s children 2015.

[5] Say L, Chou D, Gemmill A, Tunçalp Ö, Moller A-B, Daniels J, Gülmezoglu AM, Temmerman M, Alkema L (2014). Global causes of maternal death: a WHO systematic analysis. Lancet Glob Health.

[6] Vice Primatrue Charge de Sante Publique, UNFPA, UNICEF, WHO, AMDD, MSIS (2010). Evaluation des besoins en matiere de soins obstetricaux et neonatals d’urgence à Madagascar, Rapport final, Mars 2010.

[7] WHO recommendations: Uterotonics for the prevention of postpartum hemorrhage. Geneva: World Health Organization; 2018. (Licence: CC BY-NC-SA 3.0 IGO). 30645062

[8] Smith JM, Currie S, Cannon T, Armbruster D, Perri J (2014). Are national policies and programs for prevention and management of postpartum hemorrhage and preeclampsia adequate? A key informant survey in 37 countries. Glob Health Sci Pract.

[9] Braddick L, Tuckey V, Abbas Z, Lissauer D, Ismail K, Manaseki-Holland S, Ditai J, Stokes T (2016). A mixed-methods study of barriers and facilitators to the implementation of postpartum hemorrhage guidelines in Uganda. Int J Gynecol Obstet.

[10] Oladapo OT, Akinola OI, Fawole AO, Adeyemi AS, Adegbola O, Loto OM, Fabamwo AO, Alao MO, Sotunsa JO, Nigerian AMTSL Group (2009). Active management of third stage of labor: evidence versus practice. Acta Obstet Gynecol Scand.

[11] Mfinanga GS, Kimaro GD, Ngadaya E, Massawe S, Mtandu R, Shayo EH, Kahwa A, Achola O, Mutungi A, Knight R, Armbruster D, Sintasath D, Kitua A, Stanton C (2009). Health facility-based active management of the third stage of labor: findings from a national survey in Tanzania. Health Res Policy Sys.

[12] Vivio D, Fullerton JT, Forman R, Mbewe RK, Musumali M, Chewe PM (2010). Integration of the practice of active management of the third stage of labor within training and service implementation programming in Zambia. J Midwifery Womens Health.

[13] JHPIEGO (2011). Quality of care of the prevention and management of common maternal and newborn complications in health facilities in Madagascar.

[14] Schack SM, Elyas A, Brew G, Pettersson KO (2014). Experiencing challenges when implementing Active Management of Third Stage of Labor (AMTSL): a qualitative study with midwives in Accra, Ghana. BMC Pregnancy Childbirth.

[15] Datta S, Mullainathan S (2014). Behavioral design: a new approach to development policy. Rev Income Wealth.

[16] Annuaire des Statistiques du Secteur Santé de Madagascar Année 2016. Madagascar: Ministère de la Santé Publique; 2016 p. 108.

[17] Smith J, Banay R, Zimmerman E, Caetano V, Musheke M, Kamanga A (2020). Barriers to provision of respectful maternity care in Zambia: results from a qualitative study through the lens of behavioral science. BMC Pregnancy Childbirth.

[18] Braun V, Clarke V (2006). Using thematic analysis in psychology. Qual Res Psychol.

[19] O’Brien BC, Harris IB, Beckman TJ, Reed DA, Cook DA (2014). Standards for reporting qualitative research: a synthesis of recommendations. Acad Med.

[20] Sendhil M, Shafir E. Scarcity: Why having too little means so much: Macmillan; 2013.

[21] Norman DA. Some observations on mental models. In: Mental models: Psychology Press; 1983. p. 15–22.

[22] World Health Organization (WHO) (2017). Updated WHO recommendations on tranexamic acid for the treatment of postpartum haemorrhage.

[23] Prata N, Morris L, Mazive E, Vahidnia F, Stehr M. Relationship between HIV risk perception and condom use: evidence from a population-based survey in Mozambique. Int Fam Plan Perspect. 2006;32(4):192–200. 10.1363/3219206. 10.1363/3219206 17237016

[24] Maharaj P, Cleland J (2005). Risk perception and condom use among married or cohabitating couples in KwaZulu-Natal, South Africa. Int Fam Plan Perspect.

[25] Natarajan A, Ahn R, Nelson BD, Eckardt M, Kamara J, Kargbo S, Kanu P, Burke TF (2016). Use of prophylactic uterotonics during the third stage of labor: a survey of provider practices in community health facilities in Sierra Leone. BMC Pregnancy Childbirth.

[26] Higgins ET, Liberman N (2018). The loss of loss aversion: paying attention to reference points. Shavitt S, editor. J Consum Psychol.

[27] Bandura A (1986). The explanatory and predictive scope of self-efficacy theory. J Soc Clin Psychol.

[28] Cantillon P, Sargeant J (2008). Giving feedback in clinical settings. BMJ..

[29] Veloski J, Boex JR, Grasberger MJ, Evans A, Wolfson DB (2006). Systematic review of the literature on assessment, feedback and physicians’ clinical performance: BEME guide no. 7. Med Teach.

[30] Bartlett L, Cantor D, Lynam P, Kaur G, Rawlins B, Ricca J, Tripathi V, Rosen HE, on behalf of the Quality of Maternal and Newborn Care Study Group of the Maternal and Child Health Integrated Program (2015). Facility-based active management of the third stage of labour: assessment of quality in six countries in sub-Saharan Africa. Bull World Health Organ.

[31] Croskerry P (2013). From mindless to mindful practice — cognitive bias and clinical decision making. N Engl J Med.

[32] Byrne A (2013). Mental workload as a key factor in clinical decision making. Adv Health Sci Educ.

[33] Osheroff JA, Teich JM, Levick D, Saldana L, Velasco FT, Sittig DF (2012). Improving outcomes with clinical decision support: an Implementer’s guide.

