# Supplementary material for: Barriers inhibiting effective detection and management of postpartum hemorrhage during facility-based births in Madagascar: findings from a qualitative study using a behavioral science lens
Source: BMC Pregnancy Childbirth. 2021 Apr 22;21:320. doi: 10.1186/s12884-021-03801-w (PMC8063356; doi:10.1186/s12884-021-03801-w)
Supplement: Supplementary file 1 — Additional file 1. Conversation guides for interviews with providers, supervisors, postpartum women, and community health workers. [file 12884_2021_3801_MOESM1_ESM.docx]

**Breakthrough RESEARCH Provider Behavior Change Activity**

**Adherence to Best Practices for Complications Management During Facility-Based Birth**

**Conversation Guide for Provider**

**To begin, I’d like to learn a bit more about you.**

1. Could you talk to me a bit about your role, you are a midwife correct?
   1. How long have you been a midwife?
   2. What made you choose this profession?
   3. What are your main responsibilities?
   4. What part of your work do you like best? Could you describe a time when you felt very happy about your work?
   5. What part of your work do you like least? Could you describe a time when you did not feel good about your work?

**Now I would like to ask you a few questions about your work as a midwife.**

1. How do you know you are doing a good job as a midwife?
2. Do you do anything to prepare for deliveries?
   1. What do you do? What materials do you prepare?
   2. When do you prepare?
   3. Are you always able to prepare? When might you not be able to prepare?
   4. Is anyone able to help you prepare? What is their role?
   5. Does anything happen if you are not able to prepare before starting to attend deliveries and see patients?
3. Please walk me through what you do during a delivery, starting with when the woman walks into the facility to the moment when she leaves.
   1. At what point before or during labor do most women arrive to the facility?
   2. What do you do at that stage?
   3. Is there anything you ask women about when they arrive? What?
   4. Do women typically come alone or accompanied? Who do women bring as companions?
   5. What are the most important tasks you conduct during a delivery?
   6. What other tasks do you conduct? (Probe: partograph, blood pressure, fetal heart, etc.)
   7. What tasks might you conduct *only* if you have extra time?
   8. How do you remember the different tasks that you must complete during the whole delivery process? What happens if you forget a task?
   9. When might you monitor a patient during delivery? How often might you be able to monitor the patient?
   10. When might you measure a patient’s blood pressure? When else? Why then?
   11. Do you typically work alone or do other people work in the ward with you? When?
4. During delivery, when might a patient call for you or another provider?
   1. What would they need?
   2. How would you respond?
5. How do you know if the labor is progressing normally?
   1. Do you know if a labor is not progressing normally? How might you know this?
   2. What might you do if the labor is not progressing normally?
   3. When might you refer a patient to another facility?
   4. To refer a patient to another facility what is the process?
   5. Are there any challenges when you refer a patient to a facility? What?
6. What are you most concerned about during a difficult delivery?
   1. What do you do when you realize it’s going to be a difficult or dangerous delivery?
   2. What might you tell the woman to do?
7. If you see that a woman’s blood pressure is high, what do you do?
   1. If her blood pressure does not decrease, then what do you do?
8. After a baby is born, what does a midwife do? What is most important?
9. Do you visit the patients once they are in the post-natal ward? How often? What do you do when you visit them?
10. What if there is bleeding post-delivery? When do you notice this? How do you notice this? How do you know that the bleeding is dangerous?
    1. What do you do if there is bleeding?
    2. What do you do if that doesn’t stop the bleeding?
11. If a woman is not listening to you during childbirth, what can you do?
    1. When might a woman not listen to you?
    2. What kind of women don’t listen to you?
    3. What do other midwives do when this happens?
    4. What do you think of this?
12. If something happened during a labor and you didn’t know what to do, what would you do?
13. What complications might arise during childbirth? (probe: prolonged/obstructed labor, pre-eclampsia/eclampsia, PPH- ask about PE/E & PPH if doesn’t mention)

*Pre-eclampsia/eclampsia:*

- 1. How do you know it’s pre-eclampsia/eclampsia?
  2. Have you had any cases of pre-eclampsia/eclampsia? Walk me through the first time if so.
  3. What do you do in this case?
  4. How do you decide what to do?
  5. Can you decide alone or do you need to discuss it with someone else? If so who and when?
  6. How did you learn what to do in this case?
  7. What do you do then? (probe: monitor progress)
  8. What if nothing changes?
  9. How common is pre-eclampsia/eclampsia in this facility?
  10. What do you think is the cause?
  11. Are certain women at more risk? Who? Why?
  12. Are there ever supplies you need for treating pre-eclampsia/eclampsia that you don’t have? What? How often does this occur? What do you do?
  13. Are you concerned if you see you have a case of pre-eclampsia/eclampsia? What concerns you?
  14. Have you ever applied magnesium sulfate? Did you face any challenges? What? Why?

*PPH: (ensure to probe about when uteronics should be applied when this is mentioned)*

1. How do you know it’s PPH?
   1. Have you had any cases of PPH? Walk me through the first time if so.
2. What do you do in this case?
3. How do you decide what to do?
   1. Can you decide alone or do you need to discuss it with someone else? If so who and when?
4. How did you learn what to do in this case?
5. What do you do then?
6. What if bleeding continues? Then what?
7. How common is PPH in this facility?
8. What do you think is the cause?
9. Are certain women at more risk? Who? Why? (Probe: nutritional status)
10. Are there ever supplies you need for treating PPH that you don’t have? What? How often does this occur? What do you do?
11. Are you concerned if you see you have a case of PPH? What concerns you?
12. Does anyone supervise or coordinate your work?
    1. Who?
    2. How often?
    3. When they come what do they do?
    4. What do they focus on?
    5. What advice or feedback do they give you?
13. How would you describe your relationship with your supervisor? How do they make you feel?
14. In the last few years, have you received any trainings?
    1. Where were they conducted?
    2. What did they cover?
    3. Have you or your colleagues done anything differently since the training?
    4. What did you do differently and why?
15. Have there been any changes at the health facility in the last year (probe: new services, staff, processes, etc.)? What changed?
16. When you think back on your education and training to become a midwife, are there things you learned that you no longer do in your job or do differently?
    1. What are these things?
    2. Are they done by someone else? How do you do them differently and why?
17. Is there oxytocin in the facility? Where is it stored?
    1. What have you been told about oxytocin?
    2. Where did you learn this from?
    3. When might you use oxytocin? When might you not use it?
18. Are there ever stock outs of materials in your facility? What materials?
    1. Are there ever stock outs of gloves?
    2. Magnesium sulfate? Hypertensive treatment?
    3. Uterotonics?
    4. Urine tests? IVs? Catheters? Sanitary pads? Tranexamic acid? Anti-shock garment?
    5. When do these stock outs occur?
    6. What do you do?

**Now I would like to ask you a few questions about other midwives and health facilities.**

1. What do people in the community say about midwives?
   1. What makes a “good” midwife?
   2. What makes a “bad” midwife?
2. What is the worst thing that could happen to a midwife?
   1. What is the best thing that could happen to a midwife?
   2. What could you do to avoid the worst thing?
   3. What could you do to achieve the best thing?
3. Is there anything that you think other midwives should do during delivery that they do not do?
   1. What is it?
   2. Have you said or done anything about it? What?
4. Is there anything that you think other midwives should not do during delivery that they do?
   1. What was it?
   2. What did you do?
5. Have you ever heard about women who were dissatisfied about their delivery at a facility?
   1. What did they say?
   2. What complaints did they have?
   3. What did they do about it?
   4. What did you do?
6. Have you ever heard about a time when a woman or baby died during childbirth?
   1. What happened (probe: general, provider)?
   2. What are the most common causes of maternal mortality?
   3. Are certain women more at risk for death? Who? Why?
   4. What do you or other midwives think you can do to avoid these instances from occurring?
7. Is there anything else you’d like to share?

**Breakthrough RESEARCH Provider Behavior Change Activity**

**Adherence to Best Practices for Complications Management During Facility-Based Birth**

**Conversation Guide for Provider Supervisors**

**To begin, I’d like to learn a bit more about you.**

1. Could you talk to me a bit about your role, what is your title and what are your main responsibilities?
   1. What made you choose this profession?
   2. What do you enjoy most about your job?
   3. What do you enjoy least?
   4. What does it mean to be a supervisor?

**Now I would like to ask you a few questions about your work as a Supervisor or Mentor.**

1. Walk me through the process of supervision.
   1. How often do you observe the work of providers or meet with providers?
   2. How do you decide who to meet with or observe?
   3. What do you do during that time?
   4. What kind of feedback do you most often provide? Anything else?
   5. What happens after the visit?
2. Does anyone supervise or coordinate your work? Who?
   1. How often do you communicate with them?
   2. How do you communicate with them?
   3. What do they ask you about?
   4. What do they provide feedback on?
3. Have there been any changes at the health facility in the last year (probe: new services, staff, processes, changes to protocol etc.)? What changed?
   1. How did the change happen?
   2. How did the providers react to this change?

**Now I would like to ask you a few questions about your work with midwives and health facilities.**

1. Could you describe a typical midwife?
   1. Where does she come from?
   2. What is she like?
   3. How would you describe your interaction with midwifes?
   4. What do you communicate about?
2. What do you think about the midwifery profession?
   1. What do people in the community say about midwives?
   2. What makes a “good” midwife?
   3. What makes a “bad” midwife?
3. What are the most important tasks that the midwife must do?
   1. Why are these the most important?
   2. What would happen if she does not comply with them?
   3. During delivery, what are the most important tasks that the midwife must do?
   4. How does a midwife remember the tasks she must do?
4. How does a midwife know if the labor is progressing normally?
   1. How does a midwife know if labor is not progressing normally?
   2. What might she do if the labor is not progressing normally?
   3. When might she refer a patient to another facility?
   4. To refer a patient to another facility, what is the process?
   5. Are there any challenges when a patient is referred to a facility? What?
5. Do providers use the partograph? Who uses it?
   1. When do they use it?
   2. What do they use it for?
   3. What is required for them to use it?
6. After a baby is born, what are the tasks the midwife should do? What is most important?
   1. What if there is bleeding?
   2. How would a provider know that the bleeding is dangerous?
   3. What would a provider do if there is bleeding?
   4. What would a provider do if that doesn’t stop the bleeding?
7. What complications might arise during childbirth? (probe: prolonged/obstructed labor, pre-eclampsia/eclampsia, PPH – ask about PE/E & PPH if doesn’t mention)

*Pre-eclampsia/eclampsia:*

1. How would a provider know it’s pre-eclampsia/eclampsia?
2. What would a provider do in this case?
3. Are there any processes in managing pre-eclampsia/eclampsia that providers often do not follow? What are these? Why do they not follow these processes?
4. Have you attended any cases of pre-eclampsia/eclampsia? What happened in that case?
5. How common is pre-eclampsia/eclampsia in this facility?
6. What do you think is the cause?
7. Are certain women at more risk? Who? Why?
8. Are there ever supplies that providers need for treating pre-eclampsia/eclampsia that are not available? What? How often does this occur?

*PPH*:

1. How would a provider know it’s PPH?
2. What would a provider do in this case?
3. Are there any processes in managing PPH that providers often do not follow? What are these? Why do they not follow these processes?
4. Have you attended any cases of PPH? What happened in this case?
5. How common is PPH in this facility?
6. What do you think is the cause?
7. Are certain women more at risk? Who? Why?
8. Are there ever supplies that providers need for treating PPH that are not available? What? How often does this occur?
9. Have you received any trainings in the last year or two? What?
10. Have any trainings have been given to midwives in the last year or two? What?
    1. Where were these trainings given?
    2. Who gave the training?
    3. What was the training about?
    4. How was it structured? How long was it?
    5. What was the purpose?
    6. What happened after the training?
11. How skilled or knowledgeable do you think the midwife is?
    1. What makes you think that?
    2. Are there other providers who are better? Who?
    3. Why are they better?
12. If a woman is not listening to the provider during childbirth, what can a provider do?
    1. When might a woman not listen to the provider?
    2. What do most providers do?
    3. What do you think of this?
13. Is there anything that you think the midwifes should do during delivery that they do not do?
    1. What is it?
    2. Have you said or done anything about it? What?
14. Is there anything that you think the provider should not do during delivery that they do?
    1. What was it?
    2. What did you do?
15. Are there ever any stock outs of materials in your facility? What materials?
    1. Are there ever stock outs of gloves?
    2. Magnesium sulfate? Hypertensive treatment?
    3. Uterotonics?
    4. Urine tests? IVs? Catheters? Sanitary pads? Tranexamic acid? Anti-shoc garment?
    5. When do these stock outs occur?
    6. What do providers do?
16. Could you describe the process of getting oxytocin in the facility?
    1. Where does it come from? How often does it arrive? How does it arrive?
    2. What happens once it arrives? Where is it stored?
    3. Is there anything you would change about this process?

**Now I would like to ask you a few questions about the experience of your patients.**

1. If a woman is experiencing pain during childbirth, is there anything that a provider can do?
   1. What might a provider do?
   2. When might the provider do this? For whom?
   3. How would the patient respond?
2. Do you feel that the provider/midwife treats all women the same or are there differences?
   1. Who receives the best treatment?
   2. Who receives the worst treatment?
   3. Have you ever been seen by a midwife?
   4. How do you feel that you were treated by a midwife? Why?
   5. Have you ever seen others be mistreated? Who was mistreated?
   6. What happened?
   7. If a woman wants to be treated well by a provider, are there things she might do? What might she do?
3. What do you think would make a mother “difficult” during labor?
   1. What kind of women might be difficult?
   2. Why might they be difficult?
4. Do you ever receive complaints from patients who are dissatisfied with the care they have received?
   1. What kind of patients complain?
   2. Who do you hear this from?
   3. What are the complaints?
   4. What is done?
   5. How does the provider react?
5. Have you ever heard about a time when a woman or baby died during childbirth?
   1. What happened (probe: general, provider)?
   2. What are the most common causes of maternal mortality?
   3. What do providers think they can do to avoid these instances from occurring?
6. Is there anything else you’d like to share?

**Breakthrough RESEARCH Provider Behavior Change Activity**

**Adherence to Best Practices for Complications Management During Facility-Based Birth**

**Conversation Guide for Post-Partum Women**

**To begin, I’d like to learn a bit more about you.**

1. How old are you?
2. Are you married?
3. Do you have children?
   1. How many children do you have?
   2. How old are they?
4. Where were you born? Are you from this community?

**Now I would like to ask you a few questions about the last time you gave birth.**

1. How long ago did you deliver you last child?
   1. Where did you deliver your last child?
   2. What did you consider when making this decision?
   3. Where did you deliver the child before that?
   4. What did you consider when making this decision?
2. Before your last labor, did anyone explain the process of labor to you?
   1. What was explained to you about labor?
   2. Who explained this to you?
   3. What did you expect?
3. Did you come to the clinic/place for delivery with anyone else or were you alone? Who came?
   1. Did anyone else come to stay with you while you were there? Who?
   2. When did they arrive?
   3. What did they do while they were there? What did you speak about?
4. I would like you to think about when you first arrived to the clinic/place of delivery when you were in labor. When you arrived and sat down with the provider, can you please explain to me what happened?
   1. Did you have any questions for the provider?
   2. What did you ask?
   3. How did you feel with the provider?
   4. What made you feel this way?
   5. Did the provider talk to you about danger signs?
   6. What did she explain?
   7. How did you respond?
5. I would now like to ask you about when you were in labor. Can you describe your experience delivering your last child to me?
   1. How did it go?
   2. Did you ask the provider any questions? What did you ask?
   3. Did you request anything from the provider? What did you request?
   4. How did they respond?
   5. Did you (or your companion) ever call the provider? What were you calling them for?
   6. Did they come? How quickly did they come?
   7. Was the delivery painful? How painful was the delivery? Was it more or less painful than you anticipated? What did you do when you were in pain?
   8. Did the provider do anything to make you feel better? What did they do?
   9. Did anyone else in the facility help during your delivery? Who assisted?
   10. What did they do?
   11. Did you have any challenges during the delivery? What challenges? What happened?
   12. Did the provider talk to you about any problems during labor? What did they say?
6. After you gave birth, what did the provider do?
   1. Did she explain anything to you?
   2. Did you have any questions? What did you ask?
   3. How did she respond?
7. How long did you stay in the facility after giving birth?
   1. What happened during that time?
   2. How often did you see the provider?
   3. What did she do during those times?
   4. Did she explain anything to you?
8. Is there anything that you thought the provider should have done or that you would have liked them to do during the delivery that they did not do?
   1. What was it?
   2. What did you do?
9. Is there anything that you thought the provider should **not** have done during the delivery that they did?
   1. What was it?
   2. What did you do?
10. During labor did the provider ask you to do something that you did not want to do?
    1. What was it?
    2. What happened (probe: you, provider, etc.)?
    3. How did you feel?
11. How skilled or knowledgeable did you think the provider was?
    1. What made you think that?
    2. Would you have preferred a different provider? Why?
    3. Who would you have preferred? What would be different with them?
12. During the delivery process, what was most important to you?
    1. What are the reasons this was most important?
    2. Was anything else very important to you?
    3. What was it?
13. How did you feel about your delivery overall? How was the experience?
    1. Why?
    2. Did you tell anyone about your experience afterwards (probe: family, friends, provider, others)?
    3. What did you say?
    4. How did they respond?
14. Would you return to the same facility for a future delivery?
    1. Why or why not?
    2. Would you recommend this facility to a friend?
15. Did anything make you feel bad or disrespected during delivery?
    1. What happened?
    2. How did this make you feel?
    3. What did you do?

**Now I would like to ask you about your opinions about labor and midwives.**

1. What do people in the community say about midwives?
   1. What do you think about midwives?
   2. What makes a “good” midwife?
   3. What makes a “bad” midwife?
2. Do you feel that the provider/midwife treats all women the same or are there differences?
   1. Who receives the best treatment?
   2. Who receives the worst treatment?
   3. How do you feel that you were treated by the provider? Why?
   4. Have you ever seen others be mistreated by the provider?
   5. Who was mistreated?
   6. What happened?
   7. If a woman wants to be treated well by a provider, are there things she might do? What might she do?
3. Have you ever heard about a time when a woman or baby died during childbirth?
   1. What happened (probe: general, provider)?
4. Have you ever heard about a time when a woman complained about disrespectful care?
   1. Where did she complain?
   2. What happened?
5. Is there anything else you’d like to share?

**Breakthrough RESEARCH Provider Behavior Change Activity**

**Adherence to Best Practices for Complications Management During Facility-Based Birth**

**Conversation Guide for Community Health Workers**

**To begin, I’d like to learn a bit more about you.**

1. How old are you?
2. Are you married?
3. Do you have children?
   1. How many children do you have?
   2. How old are they?
4. Where were you born? Are you from this community?

**Now I would like to ask you a few questions about your work as a volunteer health worker.**

1. How long have you been a volunteer health worker?
   1. What made you want to be a volunteer?
   2. What are some of your roles and responsibilities?
   3. What training have you received? What topics were covered during the training?
2. Do you ever work at the health facility?
   1. When might that happen?
   2. What do you do when you are there?
3. Does anyone supervise or coordinate your work? Who?
   1. How often do you communicate with them?
   2. How do you communicate with them?
   3. What do they ask you about?
   4. What do they provide feedback on?
4. How often do you work with midwives?
   1. How would you describe your interactions?
   2. What do you communicate about?
   3. How do you feel working with her?
5. When do women in the community seek you out?
   1. What do you do in those cases?
   2. Are you ever called when a woman is in labor? When might this happen?
   3. What do you do in these cases?
6. Is it common for women to deliver in the home?
   1. Why do you think this occurs?
   2. Does anyone help with these deliveries?
   3. What happens if a woman has a problem during delivery?
   4. What kinds of problems do women have?
   5. Are problems during delivery common in the community? What problems? Why?
   6. Do women fear complications during delivery?
   7. When might they fear complications?
   8. What complications do they fear and what do they believe is their cause?

**Now I would like to ask you a few questions about the work of midwives at the health facility and the experience of women in your community.**

1. During delivery, what are the most important tasks that the midwife must do?
   1. What other tasks is the midwife responsible for?
2. How does a midwife know if the labor is progressing normally?
   1. Does a midwife know if labor is not progressing normally? How might she know?
   2. What might she do if the labor is not progressing normally?
3. What kind of complications might arise during childbirth?
   1. Does the midwife know if there is a complication? How does she know?
   2. What might she do in that situation?
   3. Are there times when a midwife would call for assistance?
   4. What women in the community say about complications in the facility? Do they think they are common?
   5. What do people believe is the cause of complications in these cases?
4. What do you think would make a mother “difficult” during labor?
   1. What kind of women might be difficult?
   2. Why might they be difficult?
5. What do you think about the midwifery profession?
   1. What do people in the community say about midwives?
   2. What do you think about midwives?
   3. What makes a “good” midwife?
   4. What makes a “bad” midwife?
6. Do you feel that the provider/midwife treats all women the same or are there differences?
   1. Who receives the best treatment?
   2. Who receives the worst treatment?
   3. Have you ever been seen by a midwife?
   4. How do you feel that you were treated by the provider? Why?
   5. Have you ever seen others be mistreated by a midwife? Who was mistreated?
   6. What happened?
   7. If a woman wants to be treated well by a provider, are there things she can do? What might she do?
7. If a woman is not listening to the provider during childbirth, what can a provider do?
   1. When might a woman not listen to the provider?
   2. What do most providers do?
   3. What do you think of this?
8. When women scream during labor, what happens?
   1. What does the provider do?
   2. What do you think of this?
9. Is there anything that you think the midwifes should do during delivery that they do not do?
   1. What is it?
   2. Have you said or done anything about it? What?
10. Is there anything that you think the provider should not do during delivery that they do?
    1. What was it?
    2. What did you do?
11. How skilled or knowledgeable did you think the midwife is?
    1. What makes you think that?
    2. Are there other providers who are better? Who?
    3. Why are they better?
12. Have you ever heard about women who were dissatisfied about their delivery at a facility?
    1. What did they say?
    2. What complaints did they have?
    3. Did they do anything about it?
    4. Did you do anything?
13. Have you ever heard about a time when a woman or baby died during childbirth?
    1. What happened (probe: general, provider)?
    2. What are the most common causes of maternal mortality?
    3. Are certain women more at risk for death? Who? Why?
    4. What do you think midwives can do to avoid these instances from occurring?
14. Is there anything else you’d like to share?
